# Facilitating animacy perception by manipulating stimuli exposure time

**DOI:** 10.3389/fpsyg.2022.1017685

**Published:** 2023-01-12

**Authors:** Toshiki Saito, Kosuke Motoki, Rui Nouchi, Motoaki Sugiura

**Affiliations:** ^1^School of Fundamental Science and Engineering, Waseda University, Tokyo, Japan; ^2^Japan Society for the Promotion of Science, Tokyo, Japan; ^3^Department of Management, The University of Tokyo, Tokyo, Japan; ^4^Institute for Development and Ageing and Cancer, Tohoku University, Sendai, Japan

**Keywords:** animacy perception, visual attention, gaze manipulation, mere exposure, orienting behavior

## Abstract

Animacy perception—discriminating between animate and inanimate visual stimuli—is the basis for engaging in social cognition and for our survival (e.g., avoiding potential danger). Previous studies indicate that factors in a target, such as the features or motion of a target, enhance animacy perception. However, factors in a perceiver, such as the visual attention of a perceiver to a target, have received little attention from researchers. Research on judgment, decision-making, and neuroeconomics indicates the active role of visual attention in constructing decisions. This study examined the role of visual attention in the perception of animacy by manipulating the exposure time of targets. Among Studies 1a to 1c conducted in this study, participants saw two face illustrations alternately; one of the faces was shown to be longer than the other. The participants chose the face that they considered more animated and rounder. Consequently, longer exposure time toward targets facilitated animacy perception and preference rather than the perception of roundness. Furthermore, preregistered Study 2 examined the underlying mechanisms. The results suggest that mere exposure, rather than orienting behavior, might play a vital role in the perception of animacy. Thus, in the reverse relationship between visual attention and animacy perception, animate objects capture attention—attention results in the perception of animacy.

## 1. Introduction

Animacy perception, which distinguishes animate from inanimate visual stimuli (Rutherford and Kuhlmeier, 2013), is a necessary component of social interaction. Evidence shows that such perceptions emerge even in infancy (Leslie, 1982; Gergely et al., 1995; Rochat et al., 1997) but are disrupted by developmental disorders (Rutherford et al., 2006) and amygdala damage (Heberlein and Adolphs, 2004).

Previous research on factors driving the perception of animacy mainly focused on the properties of target stimuli, such as human-like appearances (e.g., a face) and motion (e.g., interactive motion between two geometric shapes). For instance, people consider an object comparatively more animate when the object has unique human features such as eyes and mouth (Looser and Wheatley, 2010), intelligence (Bartneck et al., 2009), and facial expressions of happiness (Bowling and Banissy, 2017; Krumhuber et al., 2019; Saito et al., 2022). Furthermore, individuals consider moving objects more animate when the motion seems to have specific goals, such as chasing and helping (Heider and Simmel, 1944; Rochat et al., 1997; Castelli et al., 2000; Scholl and Tremoulet, 2000; Tremoulet and Feldman, 2000; Kuhlmeier et al., 2003).

Although most previous research has shown that factors in a target (e.g., human-like features of targets) play crucial roles in animacy perception, these factors do not necessarily facilitate it. According to the uncanny valley theory (Mori et al., 2012), inanimate objects (e.g., robots) resemblance to humans increases the perception of animacy. However, when the resemblance reaches a certain point, it provokes uncanny or strange feelings and hinders the perception. The uncanny valley theory suggests the importance of focusing not only on factors in a target but also on factors in a perceiver. However, minimal extant literature focuses on the factors (e.g., knowledge and mental state of participants) in animacy perception. For example, beliefs about the origin of moving objects (i.e., humans or robots; Cross et al., 2016) and the state of participants (i.e., loneliness) affect animacy perception (Powers et al., 2014). Thus, it is also essential to focus on factors affecting animacy perception in perceivers.

Notably, attention, which can be counted as a factor in perceivers, has a critical relationship with animacy perception and might be a causal effect in animacy perception. Previous studies show that animate objects capture attention (Pratt et al., 2010; Yang et al., 2012; Jackson and Calvillo, 2013; Calvillo and Jackson, 2014). For example, when individuals are tasked with finding a category exemplar and are unexpectedly exposed to either an animate or inanimate object, they are more likely to notice the animate object (Calvillo and Jackson, 2014). Thus, the authors concluded that these findings reflect that detecting animate objects is vital in ancestral hunter-gatherer environments and is consistent with the animate-monitoring hypothesis (New et al., 2007). As mentioned above, animate objects attract attention. However, is there also a reversal relationship between them? Specifically, does attracting attention lead to animacy perception? This prediction could be the case considering recent research on judgment and decision-making.

The growing body of research on judgment, decision-making, and neuroeconomics highlights the crucial role of visual attention in decision-making (Armel et al., 2008; Krajbich et al., 2010; Glöckner and Herbold, 2011; Krajbich and Rangel, 2011; Krajbich et al., 2012; Orquin and Loose, 2013; Cavanagh et al., 2014; Thomas et al., 2019). In particular, the attentional drift-diffusion model (aDDM), proposed by Krajbich and his peers, incorporates the role of visual attention in traditional decision-making models (i.e., the drift-diffusion model; Krajbich et al., 2010; Krajbich and Rangel, 2011; Krajbich et al., 2012; Krajbich, 2019). The aDDM is a decision-making model assuming that the evidence of an item for reaching a decision is amplified when the item receives more attention. Notably, assuming that visual attention modulates the accumulation of evidence to reach a threshold to decide, decision times and choices can accurately be predicted (Krajbich et al., 2010). Previous neural studies present supportive evidence that there was neural activity related to fixation-dependent value coding but did not examine the validity of aDDM (Lim et al., 2011; McGinty et al., 2016). Furthermore, numerous behavioral studies have shown that a longer gaze duration toward one option results in a higher choice probability for that option (Shimojo et al., 2003; Armel et al., 2008; Krajbich et al., 2010, 2012; Saito et al., 2017, 2020; Thomas et al., 2019; Motoki et al., 2021). Moreover, behavioral studies indicate the causal role of attention in decision-making by manipulating the gaze toward options and that the probabilities of choices have changed (Shimojo et al., 2003; Armel et al., 2008; Pärnamets et al., 2015). As mentioned above, visual attention plays a crucial role in decision-making. By employing this perspective, we sought to specify factors in a perceiver affecting animacy perception in the current study.

Gaze manipulation does not always bias decision-making (Shimojo et al., 2003). Though, it is reported that there is a consistent effect on simple perceptual choice (Tavares et al., 2017). According to Shimojo et al. (2003), gaze manipulation can influence subjective (e.g., preference) rather than objective judgments. In other words, gaze manipulation is likely to influence higher-level cognition (e.g., preference) rather than low-level perception (e.g., morphological perception). Given that preference for targets contributes to an uncanny valley feeling (Wang and Rochat, 2017), animacy perception may be influenced by gaze manipulation through a preference for targets. This study directly tests this hypothesis where gaze manipulation influences the animacy perception.

Exposure duration (i.e., mere exposure effect; Zajonc, 1968) and gaze shifting (i.e., gaze orienting) may be potential mechanisms influencing the role of visual attention in animacy perception. The account of exposure duration is based on the mere exposure effect (Zajonc, 1968). Specifically, the more people look at a stimulus, the more they like it. It has also been assumed that gaze orienting is a precursor to higher-level cognition (e.g., preferences; Shimojo et al., 2003; Simion and Shimojo, 2006; Simion and Shimojo, 2007). More prolonged exposure durations with orientation (i.e., gaze shifting) can induce a preference shift. In contrast, longer exposure durations without orientation do not result in a preference shift (Shimojo et al., 2003). We further elucidated the potential underlying mechanisms by manipulating exposure duration and gaze orientation.

This study examines whether stimuli exposure time influences the perception of animacy. In particular, we investigated whether the manipulation influenced high-level perceptions, animacy perception (Study 1a), and preference (Study 1b). Additionally, we examined the effect on roundness judgment (Study 1c), which we considered low-level perception. We expected high-level cognition (i.e., animacy and preference), rather than low-level perception, would be biased by gaze manipulation. Furthermore, Study 2 examined the underlying mechanisms by separating the factors of gaze manipulation into exposure duration and arbitrary eye movements. As we mentioned above, it is reported that arbitrary eye movement is necessary for biasing high-level cognition (Shimojo et al., 2003). However, contradicting findings reported that extended exposure duration, regardless of gaze orientation, biased decision-making (e.g., Nittono and Wada, 2009; Bird et al., 2012). Thus, we examined which factor of gaze manipulation, exposure duration, or arbitrary eye movements influence animacy perception in Study 2.

## 2. Study 1a to 1c

Study 1a examined the effects of gaze manipulation on animacy perception. The participants viewed two facial images with artificial features and then chose the image perceived as more animate. While viewing the images, participants’ eye movements were manipulated using the paradigm of a previous study (Shimojo et al., 2003). Study 1b was designed to replicate the effect of gaze manipulation on preference judgment (Shimojo et al., 2003) in the current experimental procedure. The procedure was similar, except that it made participants choose their preferred facial images. Study 1c was designed to confirm the specificity of gaze manipulation for both preference and animacy perception. The procedure was almost the same, except that it required participants to choose a rounder facial image.

### 2.1. Methods

#### 2.1.1. Participants

To the best of our knowledge, because no prior study has examined the effect of exposure time on animacy perception, we did not formally calculate the sample size for Studies 1a to 1c. We recruited university students who participated in each study during the 1st wave of the recruitment period. Finally, 43 participants for Study 1a (11 women, 32 men; mean age, 20.78; SD of age, 1.38), 61 participants for Study 1b (20 women, 41 men; mean age, 21.13; SD of age, 2.75), and 29 participants for Study 1c (12 women, 17 men; mean age, 21.41; SD of age, 1.37) were selected. We considered those sample sizes (i.e., 29–61) almost sufficient to detect the effect given previous studies’ sample sizes ranged from 10 to 100 (Shimojo et al., 2003; Armel et al., 2008). The participants were all university students recruited *via* a university bulletin board and mailing list. After completing the study, participants received a small monetary compensation for their participation. This study was approved by the ethics committees of Tohoku University (Number: UMIN000025712) and Waseda University [Number 2019-357(1)] and conducted per the Declaration of Helsinki. For each study, the participants gave their free and informed consent.

#### 2.1.2. Stimuli

**Figure 1:**
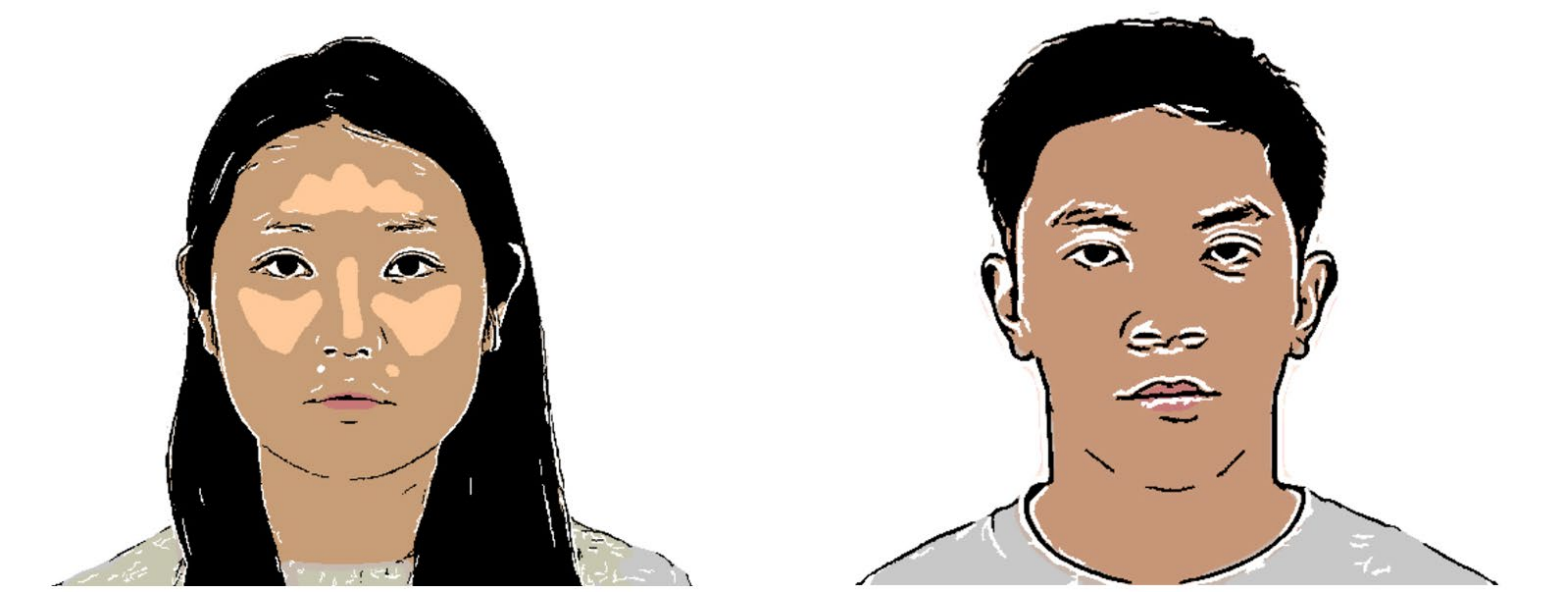
Examples of modified face images used in the studies. Adapted with permission from Chicago Face Database, available at https://www.chicagofaces.org/ (Ma et al., 2015).

In this study, we used 40 pairs of facial images (20 female and 20 male face pairs). To create these images, we selected 45 male and 45 female faces from the Chicago Face Database (Ma et al., 2015). All facial images displayed no emotional expression (i.e., neutral expression). The images depicted real human faces and might cause a ceiling effect on animacy perception that prevented the effects of gaze manipulation. Therefore, we modified these images to add artificial features using non-photorealistic rendering methods (Rosin and Lai, 2015). This method produces realistic cartoons from real images of the same identity (Figure 1). The images were resized to a uniform width of 600 pixels and height of 450 pixels.

We further conducted an online pre-experiment to manipulate the attractiveness of the images using Qualtrics. We recruited 40 participants *via* Lancers^1^ and asked them to rate the attractiveness of the images on a 7-point Likert scale (1 = very unattractive to 7 = very attractive). Based on these ratings, we created 40 pairs of facial images. Stimulus codes for the exact stimuli employed are available in the online supplemental material.^2^ The average ratings of the faces in a pair were matched such that the difference in the average rating in each pair was <0.10 points. The average rating for all faces was 3.12 (SD = 0.49). The faces in a pair were also matched in terms of sex. There was an equal number of face pairs in each sex (20 male and 20 female face pairs).

#### 2.1.3. Procedure

**Figure 2:**
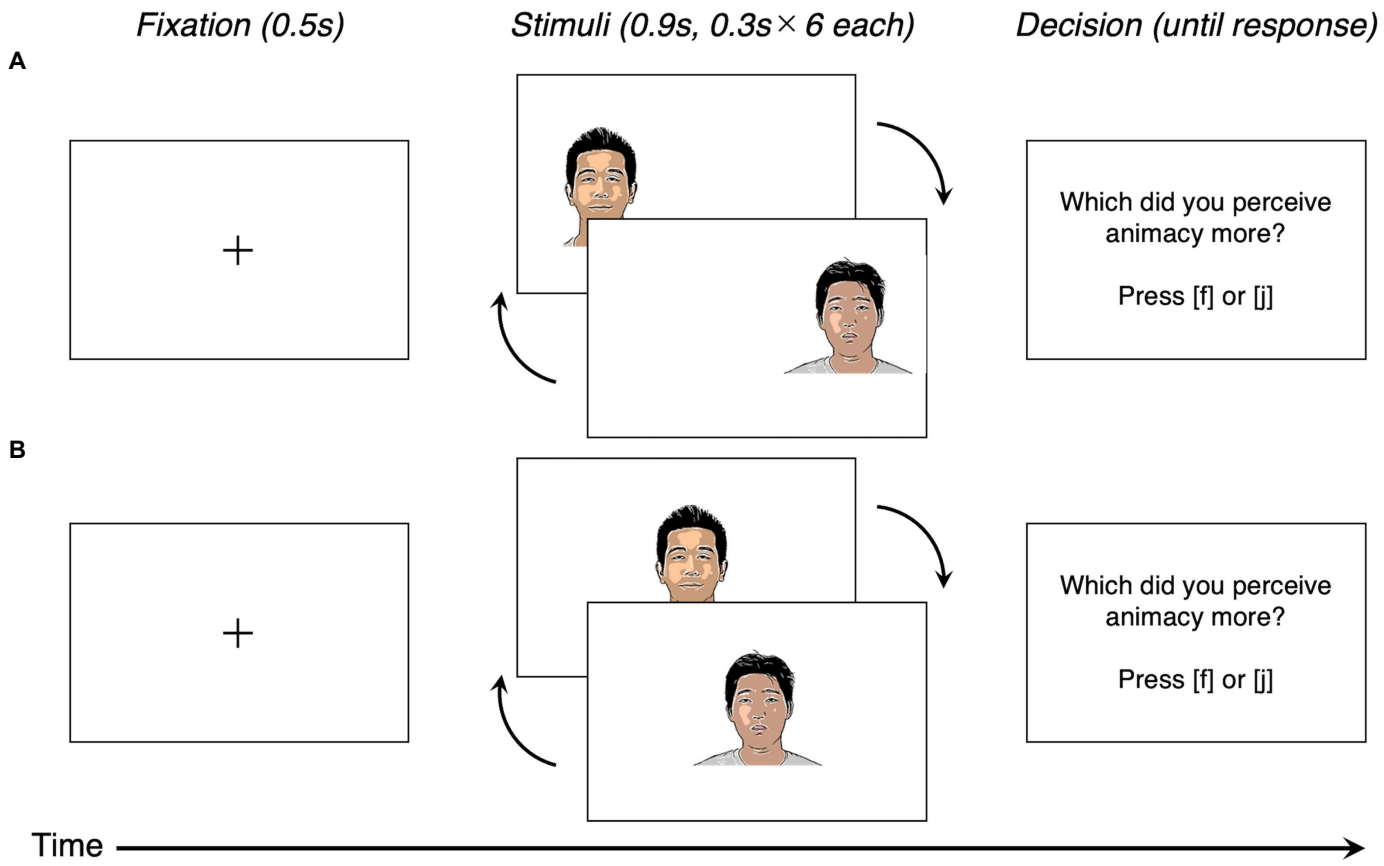
Example of task flow in Studies 1a to 1c and Study 2. Note. In Studies 1b, 1c, and 2, the instruction for decision-making was changed depending on the conditions [which did you prefer?] for Study 1b; and which did you perceive as rounder?; for Studies 1c and 2. **(A)** the arbitrary eye movement condition in Studies 1a-1c, 2 and **(B)** the fixed eye movement condition in Study 2. Adapted with permission from Chicago Face Database, available at https://www.chicagofaces.org/ (Ma et al., 2015).

We used similar experimental procedures and conducted the experiments almost concurrently. Based on a previous study (Shimojo et al., 2003), we manipulated stimuli exposure time to participants while perceiving a pair of faces (Figure 2A).

Participants completed the experiment individually on a computer (display resolution 14-inch, 1920 × 1080). The distance between the participant’s eyes and the display was approximately 60 cm. After showing the fixation cross for 500 ms, we presented each face six times to the participants. Faces alternated between the left and right halves of the screen. Therefore, participants had to shift their gaze toward the visible face on the screen. The presentation duration for each face in a pair was different, 900 ms for one face and 300 ms for another face. At one trial, one face was shown for 5400 ms (900 ms × 6 times) and another for 1800 ms (300 ms × 6 times). These durations were identical to those of the previous study (Shimojo et al., 2003). Faces that were shown longer than other faces were counterbalanced across the participants. After viewing a pair of faces, participants chose a face in which they perceived animacy more (Study 1a), preferred more (Study 1b), or perceived rounder (Study 1c) by pressing the corresponding keys. For instance, the “f” key for the left-sided face and the “j” key for the right-sided face. The reaction time was not constrained, and the order of face pair presentation was randomized across trials. The total number of trials was set to 40. Before the experiment, we explained the procedure to participants and confirmed their understanding of the instruction by asking them.

#### 2.1.4. Statistical analysis

Through Studies 1a to 1c, we used mixed logistic models to predict the choice of the target presented on the left side (1: left-sided target, 0: right-sided target), with the left-sided target shown for a long or short duration (1: shown longer, 0: shown shorter) as a fixed effect, and participants and pairs of stimuli included as a random slope and a random intercept. All analyses were conducted using the lme4 package (Bates et al., 2014) in the R software (R Core Team, 2021). Regarding the analysis in Study 1c, we excluded 12 trials in which the stimuli were not presented for the intended duration owing to technical issues. In conclusion, we analyzed the data of 28 trials from each participant in Study 1c. The data analyzed in this study were made available at the Open Science Framework.^3^

### 2.2. Results

#### 2.2.1. Study 1a (Animacy judgment)

**Figure 3:**
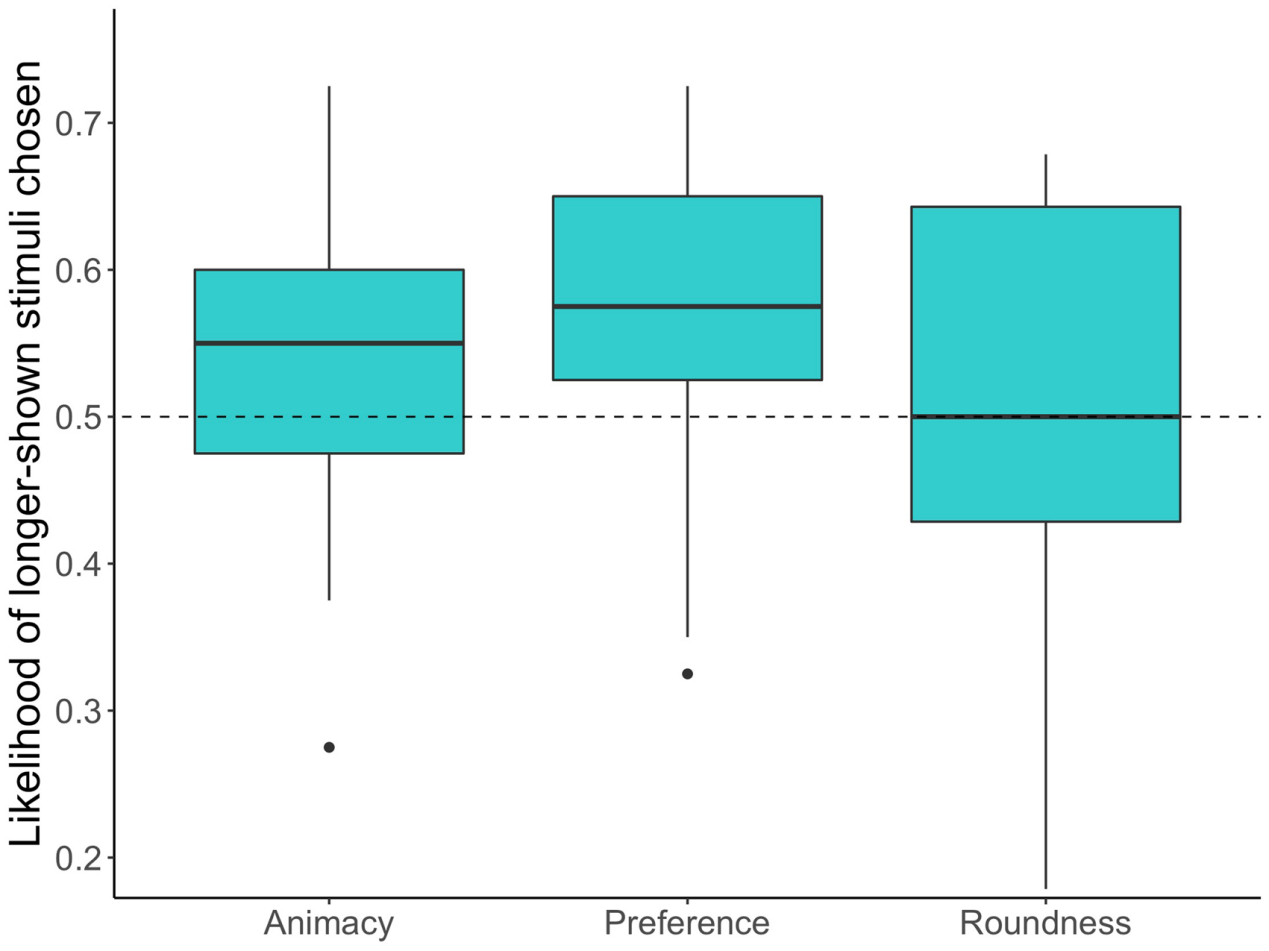
The box plots of the likelihood of the longer-shown stimuli chosen by participants across Studies 1a to 1c. The dashed line represents the chance level (50%).

The results of the analysis showed that participants tended to choose longer-shown faces as more animated faces in Study 1a (53.89, 95% CI [51.89–55.89]; *b* = 0.34, *z* = 3.19, *p* < 0.001). This result suggests that gaze bias influenced perceptions of animacy. The likelihoods of longer-shown stimuli chosen through Studies 1a to 1c are visualized in Figure 3. Table 1 further shows the details of the results.

**Table 1:** Fixed effects from the GLMM analyses through Studies 1a to 1c.

Study	Predictor	*β*	SE	*z*-value	Value of *p*	OR	95% (OR)
Study 1a (Animacy)	Intercept	−0.28	0.11	−2.57	0.010	0.76	[0.61, 0.94]	Presentation Duration	0.34	0.11	3.19	0.001	1.40	[1.14, 1.72]
Study 1b (Preference)	Intercept	−0.33	0.14	−2.41	0.016	0.72	[0.55, 0.94]	Presentation Duration	0.53	0.13	4.21	0.001	1.71	[1.33, 2.19]
Study 1c (Roundness)	Intercept	0.19	0.15	1.28	0.202	1.20	[0.91, 1.60]	Presentation Duration	−0.02	0.24	−0.08	0.936	0.98	[0.61, 1.58]

OR, odds ratio.

#### 2.2.2. Study 1b (preference judgment)

The results of the analysis revealed that participants preferred the faces that were shown longer (57.03, 95% CI [54.65–59.39]; *b* = 0.53, *z* = 4.21, *p* < 0.001). This indicates that we successfully replicated the effect of eye movement on preference judgment (Shimojo et al., 2003; Krajbich et al., 2010; Saito et al., 2017).

#### 2.2.3. Study 1c (roundness judgment)

The results of the analysis indicated that participants did not tend to choose longer-shown faces as rounder faces in Study 1c (49.88, 95% CI [46.38–53.37]; *b* = −0.02, *z* = −0.08, *p* = 0.98). This result suggests that gaze bias specifically influenced both preferences and animacy perception rather than morphological perception (i.e., roundness judgment).

### 2.3. Discussion

Through Studies 1a to 1c, we observed that gaze manipulation influences animacy and preference judgments, not roundness judgments. These findings suggest the specificity of the effect of gaze manipulation on animacy and preference perceptions and that these perceptions might be affected by gaze manipulation through the exact mechanism. However, regarding the mechanism, it is unclear what aspect of gaze manipulation we used affected the perceptions because we manipulated the presentation duration (i.e., mere exposure) and arbitrary eye movements (i.e., orienting behavior). Furthermore, we did not directly compare the effects of gaze manipulation on animacy and roundness judgment. Therefore, Study 2 was designed to address these questions.

## 3. Study 2

In Study 2, we sought to solve the issues mentioned above by directly comparing the effects of (1) present duration (i.e., mere exposure) and arbitrary eye movements (i.e., orienting behavior) and (2) animacy and roundness judgment. To specify the factors of gaze manipulation on animacy perception, we used a paradigm in which participants’ eye movements were fixed (Shimojo et al., 2003).

### 3.1. Method

#### 3.1.1. Experimental design

This study included two independent variables: the type of judgment (two levels: animacy and roundness) and gaze manipulation (two levels: arbitrary eye movement and fixed eye movement). These variables were between-participant factors. The dependent variable was the choice of stimulus.

#### 3.1.2. Participants and stimuli

We conducted a simulation-based power analysis using the SIMR package (Green and MacLeod, 2016) in R and the data from Study 1a to estimate the ideal sample size. This analysis determined the expected power to secure the fixed effect of gaze manipulation for various sample sizes. The results indicated the need for a sample size of 169 to achieve over 80% at an alpha level of 0.05. Considering that the average dropout rate of a typical web experiment is approximately 30% (Musch and Reips, 2000; Zhou and Fishbach, 2016), we determined the sample size to be 220 participants (55 participants for each condition). We recruited participants using Lancers. A total of 221 participants were recruited for the study. After excluding participants who failed the attention check, data from 205 participants (63 women, 136 men, 6 preferred not to disclose; mean age, 41.98; SD of age, 8.66) were analyzed. The participants received a small monetary compensation for their participation. This study was approved by AsPredicted.org.^5^ Further, we used the same stimuli as in Studies 1a to 1c.

#### 3.1.3. Procedure

The procedure was almost identical to those of the previous studies, except as noted in the following text. We conducted the study online through Qualtrics^6^ because it was challenging to experiment in person due to the coronavirus disease 2019 pandemic at that time (October 18–25, 2021). At the beginning of each study, participants answered a question designed to check whether they read instructions as an attention check (Oppenheimer et al., 2009). In particular, participants had to ignore the standard response format and instead provide a confirmation that they had read the instruction in the question. Then, participants were randomly assigned to one of four conditions (two types of judgment: animacy, roundness × 2, gaze manipulation: arbitrary eye movement, and fixed eye movement). In Study 2, we established a fixed eye movement condition, where the stimuli face alternated at the center of the screen (Figure 2B). Thus, the participants did not have to shift their gaze toward the visible face on the screen. After viewing each pair of faces, participants chose the face they perceived as having more animacy (animacy condition) or rounder (roundness condition) by pressing the corresponding keys. For instance, the “f” key for the face presented at last and the “j” key for the face presented before the last. In conclusion, 104 participants were assigned to the animacy judgment condition (53 in arbitrary eye movement, 51 in fixed eye movement), and 101 participants were further assigned to the roundness judgment condition (52 participants in arbitrary eye movement, 49 participants in fixed eye movement).

### 3.2. Statistical analysis

Study 2 used the preregistered analysis, which was a linear mixed model predicting the choice of one target (arbitrary eye movement condition: 1 = left-sided target, 0 = right-sided target; fixed condition: 1 = the last-presented target, 0 = before the last-presented target), with the target was shown for a long or short duration (presentation duration: 1 = shown longer, 0 = shown shorter), gaze manipulation (1 = arbitrary eye movement, 0 = fixed eye movement), types of judgment (1 = animacy, 0 = morphological perception). Further, the interactions were included as fixed effects, and participants and pairs of stimuli were included as random effects. We used the performance package (Lüdecke et al., 2021) in the R software to investigate the variance inflation factors (VIFs) and overdispersion.

In addition to the preregistered analysis, we conducted similar analyses to the previous three studies as exploratory analyses for analytical consistency across studies. For each condition, we conducted an analysis that was a mixed logistic model predicting the choice of one target (arbitrary eye movement condition: 1 = left-sided target, 0 = right-sided target; fixed condition: 1 = the last-presented target, 0 = before the last presented target), with the target shown for a long or short duration (1 = shown longer, 0 = shown shorter), as a fixed effect. Further, participants and pairs of stimuli were included as a random slope and random intercept, respectively.

### 3.3. Results

**Figure 4:**
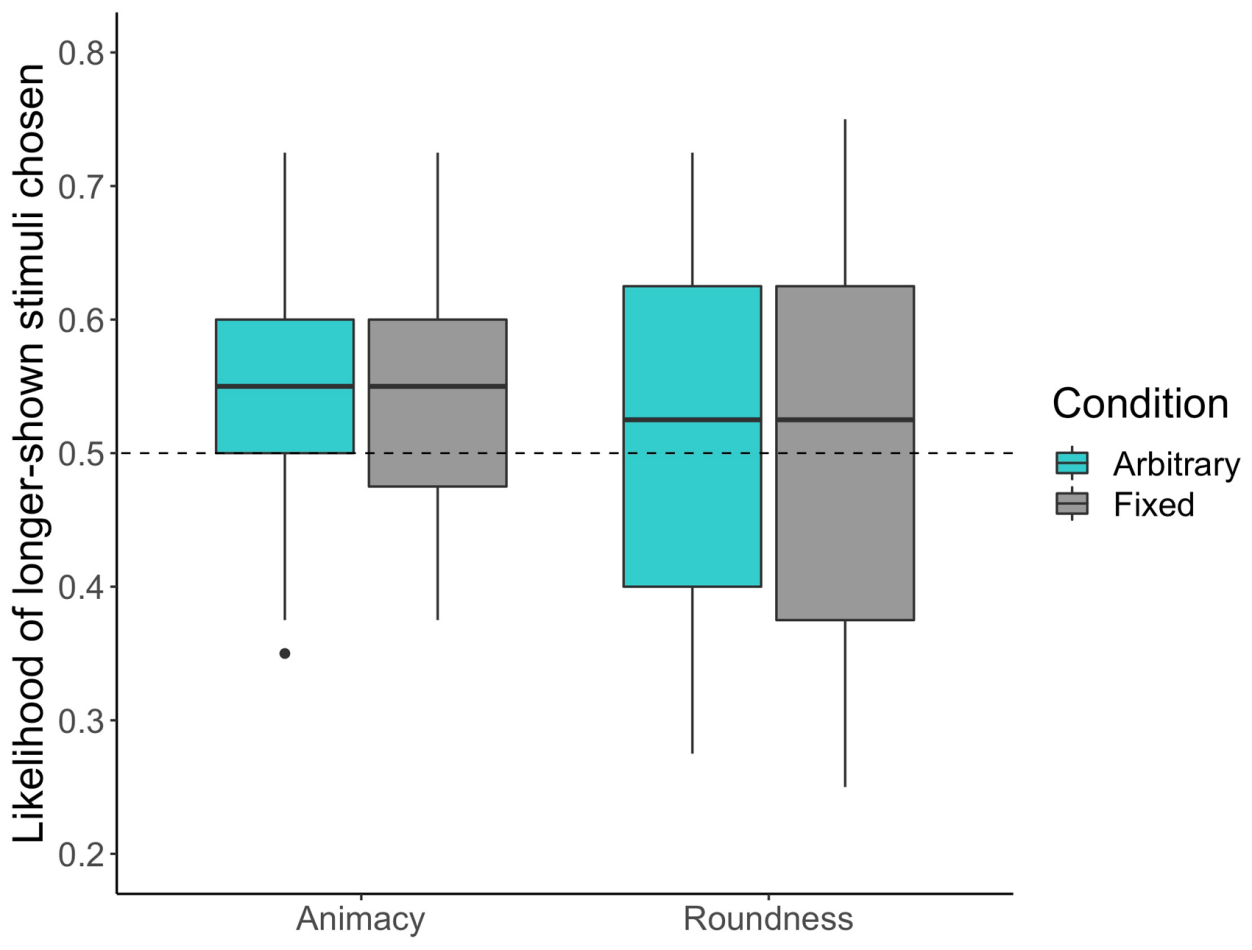
The box plots of the likelihood of the longer-shown stimuli chosen by participants in Study 2. The dashed line represents the chance level (50%).

Figure 4 shows the likelihood of choosing longer-shown faces. Regarding the preregistered analysis, we confirmed that multicollinearity was not a problem by inspecting the VIFs (VIFs < 3.91). Further, overdispersion was not a problem in the overdispersion test (*χ*^2^ = 7157.48, *p* = 1.00). The result from the preregistered analysis showed neither significant effects of presentation duration, gaze manipulation, and types of judgment nor those interactions (Table 2).

**Table 2:** Fixed effects from the registered analysis predicting the choice in Study 2.

Predictor	*β*	SE	*z*-value	Value of *p*	OR	95% (OR)
Intercept	−0.13	0.19	−0.72	0.473	0.87	[0.61, 1.26]
Presentation duration	0.30	0.28	1.08	0.282	1.35	[0.78, 2.35]
Gaze manipulation	−0.12	0.26	−0.46	0.643	0.89	[0.53, 1.49]
Type of choice	0.39	0.27	1.45	0.148	1.47	[0.87, 2.48]
Presentation duration × Gaze manipulation	0.14	0.40	0.34	0.732	1.15	[0.53, 2.50]
Presentation duration × Type of choice	−0.12	0.40	−0.30	0.762	0.89	[0.40, 1.95]
Gaze manipulation × Type of choice	−0.35	0.38	−0.93	0.353	0.70	[0.34, 1.48]
Presentation duration × Gaze manipulation × Type of choice	−0.27	0.57	−0.47	0.641	0.77	[0.25, 2.34]

OR, odds ratio.

Although we did not observe any significant results from the registered analysis, we conducted a mixed logistic model to predict the choice of one target for each condition, as in previous studies (Table 3).

**Table 3:** Fixed effects from the additional GLMM analyses of each condition in Study 2.

Type	Condition	Predictor	*β*	SE	*z*-value	Value of *p*	OR	95% (OR)
Animacy	Arbitrary	Intercept	−0.25	0.11	−2.35	0.019	0.78	[0.63, 0.96]		Presentation duration	0.42	0.15	2.82	0.005	1.52	[1.14, 2.03]	Fixed	Intercept	−0.12	0.08	−1.50	0.133	0.88	[0.75, 1.04]		Presentation duration	0.28	0.13	2.23	0.026	1.33	[1.04, 1.70]
Roundness	Arbitrary	Intercept	−0.29	0.33	−0.88	0.379	0.75	[0.40, 1.42]		Presentation duration	0.03	0.52	0.06	0.950	1.03	[0.38, 2.84]	Fixed	Intercept	0.25	0.23	1.12	0.264	1.29	[0.83, 2.01]		Presentation duration	0.21	0.35	0.59	0.558	1.23	[0.62, 2.46]

OR, odds ratio.

In the arbitrary eye movement condition, we observed that participants tended to choose longer-shown faces when choosing more animate faces (54.86, 95% CI [52.71–56.99]; *b* = 0.42, *z* = 2.77, *p* = 0.01) than when choosing rounder faces (50.48, 95% CI [48.31–52.65]; *b* = 0.05, *z* = 1.14, *p* = 0.26). In the fixed eye movement condition, we also observed that participants tended to choose longer-shown faces when choosing more animate faces (53.43, 95% CI [51.24–55.61]; *b* = 0.29, *z* = 2.28, *p* = 0.02) but not when choosing rounder faces (50.71, 95% CI [48.48–52.95]; *b* = 0.23, *z* = 0.61, *p* = 0.54).

### 3.4. Discussion

In Study 2, the preregistered analysis showed neither significant effects nor interactions between the experimental conditions. Therefore, we failed to elucidate the factors of gaze manipulation (i.e., mere exposure and orienting behavior) that influence animacy perception. However, subsequent exploratory analyses were consistent with Studies 1a to 1c, showing that gaze manipulation in arbitrary and fixed eye movement conditions influenced only animacy perception rather than the perception of roundness. These results suggest that mere exposure may be critical in facilitating animacy perception.

## 4. General discussions

Factors in a perceiver have not received sufficient attention regarding the factors that drive animacy perception. We tested whether one of the primary factors, visual attention toward stimuli, affects animacy perception. Across Studies one and two, the participants felt that cartoon faces were more animated when manipulating their gaze to look at the faces longer. This effect of biased exposure duration was also observed in preference judgments (Study 1b) rather than in lower-level perception (i.e., roundness judgments, Studies 1c and 2). Furthermore, in the preregistered online study (Study 2), it was found that arbitrary eye movements were not necessarily needed to increase animacy perception. However, exposure duration played a crucial role in influencing it.

Our results provide evidence that gazing behavior influences the perception of animacy. In this study, manipulating the exposure duration in arbitrary and fixed eye movement conditions facilitated animacy perception. This finding is inconsistent with the claim that gaze orienting is necessary to bias higher-level cognition, such as preference judgment (Shimojo et al., 2003). Instead, this finding is consistent with studies that show that gaze orienting is not a necessary condition for forming higher-level cognition but instead demonstrates that a mere exposure effect underlies biased higher-level cognition by gaze manipulation (Glaholt and Reingold, 2009; Nittono and Wada, 2009; Glaholt and Reingold, 2011; Bird et al., 2012).

There are several potential explanations for why the mere exposure effect derives animacy perceptions. First, along with aDDM (Krajbich et al., 2010), attention would have facilitated evidence of animacy and preference. Second, mere exposure may have changed several psychological constructs, as mere exposure increases familiarity and saliency (Montoya et al., 2017). Familiarity seemed to be a crucial construct in this study, given the uncanny valley theory, where unfamiliarity or strangeness hinders the perception of animacy (Mori et al., 2012). Moreover, an empirical study indicated that people attribute fundamental capacities of the mind, which is a concept strongly related to animacy, to preferred targets (Kozak et al., 2006). Examining the relationship between the mere exposure effect and animacy will likely be a pivotal issue for future work.

We have observed that the choice probabilities of longer-shown stimuli in the animacy condition were greater than chance. Nonetheless, it is important to note that in Study 2’s preregistered analysis, we could not identify the gaze manipulation components that affected animacy perception. Given that the preregistered analysis did not reveal significant effects of judgment types and gaze manipulations on the choice probabilities, we cannot conclude that gaze manipulation uniquely affected animacy perception. Instead, we need to stress that the effect of gaze manipulation on animacy perception might be limited or relatively small. The degree and the uniqueness of the gaze manipulation effect on animacy perception should be further examined in future studies.

Future work would be needed to specify the relationship between visual attention and animacy perception in detail. Firstly, it is necessary to test whether the effect of gaze manipulation occurs for completely inanimate objects (e.g., simple geometrics). The facial stimuli in the present study seemed relatively animate; therefore, it is unclear whether the gaze manipulation effect can trigger animacy perception. Thus, testing whether exposure duration facilitates the perception of animacy, even when the targets are entirely inanimate, would be an interesting direction. It is crucial to inspect the underlying mechanisms biased by gaze manipulation directly influencing animacy perception. Notably, attentional bias results in changes in the target’s characteristics, such as saliency, liking (Mrkva and Van Boven, 2020), and familiarity (Montoya et al., 2017). Therefore, future studies need to examine the psychological mechanisms that mediate the relationship between gaze manipulation and animacy perception.

This study tested whether exposure time plays a role in the perception of animacy. We found evidence that biased exposure time of targets facilitated both animacy perception and preference toward targets rather than lower-level perception (i.e., morphological judgment). The underlying mechanisms biased by gaze manipulation directly influencing animacy perception are not clear. However, our findings suggest that biasing visual attention toward targets facilitates animacy perception, possibly because mere exposure increases familiarity or preference.

## Data availability statement

The datasets presented in this study can be found in online repositories. The names of the repository/repositories and accession number(s) can be found at: https://osf.io/cr4yx/.

## Ethics statement

The studies involving human participants were reviewed and approved by the ethics committees of Tohoku University. The patients/participants provided their written informed consent to participate in this study.

## Author contributions

TS: conceptualization, methodology, investigation, software, formal analysis, writing—original draft preparation. KM: conceptualization, methodology, investigation, writing—reviewing and editing. RN: methodology, writing—reviewing and editing. MS: supervision, writing—review and editing. All authors contributed to the article and approved the submitted version.

## Funding

This research was supported by a Grant-in-Aid for JSPS Fellows (21J01224; TS) from the Japan Society for the Promotion of Science and JSPS KAKENHI 19H01760 [Grant-in-Aid for Scientific Research (B)] and JSPS KAKENHI 19H05003 [Grant-in-Aid for Scientific Research on Innovative Areas (Research in a Proposed Research Area)] (RN).

## Conflict of interest

The authors declare that the research was conducted in the absence of any commercial or financial relationships that could be construed as a potential conflict of interest.

## Publisher’s note

All claims expressed in this article are solely those of the authors and do not necessarily represent those of their affiliated organizations, or those of the publisher, the editors and the reviewers. Any product that may be evaluated in this article, or claim that may be made by its manufacturer, is not guaranteed or endorsed by the publisher.

## Footnotes

^1^ https://www.lancers.jp

^2^ https://osf.io/cr4yx/?view_only=633225a44c9f455993688e2c96ea382c

^3^ https://osf.io/cr4yx/

^5^ https://aspredicted.org/492_915

^6^ https://www.qualtrics.com/jp/
